# Phlegmatia Dolens

**Published:** 1824-09-01

**Authors:** 


### VIII.

### PHLEGMATIA DOLENS.

1. *An Essay on the Proximate Cause of Phlegmatia Dolens.* By DAVID D. DAVIS, M.D.

[Medico-Chirurgical Trans. Vol. XII. Part II.]

2. *Observations on Phlegmatia Dolens.* By JOHN W. FRANCIS, M.D. of New York.

[New York Med. and Phys. Journal, No. 1.]

3. *Observations on Cruritis, or Phlegmatia Dolens.* By DAVID HOSACK, M.D. of New York. *Ibidem.*

---

"Multùm prodesse ad intelligendas morborum causas cadaverum sectionem nemo dubitat:—interim tamen magnâ cautelâ hìc opus est, ne pro morbi causa habeatur illud, quod potius morbi effectus est: multa enim in cadavere inveniuntur mutata per morbum ipsum, quæ non præexstiterant ante morbum."—*Van Swieten's Commentaries on Boerhaave's Lectures, Sect.* 1041.

---

WE recommend to the serious consideration of every pathologist the admirable and memorable remark of Van Swieten, prefixed as a motto to our present article. We are convinced that there is not a more common mistake in pathological researches than that of setting down effects as causes. In the theories of fevers, broached and maintained in various countries, how often have the traces of inflammation found after death been pronounced the cause and not the consequence of the pyrexial phenomena?—has not the watery effusion in hydrocephalus acutus been set down as the primary state of the head, instead of the mere consequence of inflammatory action in the vessels of the brain?—These examples might be multiplied to a great extent. The perusal of Dr. Davis's paper has induced us to think that, founded as his theory is upon facts and dissections, apparently the most accurate and authentic, yet that he may have fallen into the same error as many of his illustrious predecessors. But this is prejudging the question. He shall speak for himself.

Up to Dr. Davis's time, four different theories of phlegmatia dolens have been maintained, with more or less plausibility, and grounded on some of the obvious phenomena of the disease. Mauriceau's Hypothesis was *Metastasis of the Lochia*—Puzos attributed the disease to a *translation or a depot of milk*, which has maintained considerable credit in the continental schools. Mr. White and others espoused the doctrine of *obstruction or disease of the lymphatics*—while Dr. Hull, of Manchester, in his Essay on Phlegmatia Dolens, maintains that "*the prox-*

*imate cause consists in an inflammatory affection, producing suddenly a considerable effusion of serum and coagulable lymph from the exhalents into the cellular membrane of the limb.*" The seat of the disease he believes to be in the muscles, cellular membrane, and inferior surface of the cutis. "In some cases, he observes, the inflammation may be communicated to the large blood-vessels, nerves, the lymphatic glands, and glands imbedded in them." From these passages it is evident that Dr. Hull has left only the *bones* as new or unoccupied ground for future investigators. But then the question of *priority* of structure invaded by the disease is still open for discussion, and this is the field pitched upon by Dr. Davis. Dr. D. keenly enough remarks that this "capacious theory" of Dr. Hull is not attempted to be founded on any evidence derived from anatomical examination—and here, it must be confessed, is an insurmountable defect—a defect from which Dr. Davis's own theory will not, perhaps, be found quite free, as we shall show in the sequel. As an excuse for Dr. Hull, it is conceded that no *post mortem* evidence existed at the time he published—if we except a case recorded by Gottfrey Zinn, in the year 1753, to be found in the second volume of the Commentaries of the Royal Society of Gottingen. This case is alluded to by Dr. Hull, but he is unwilling to consider it as a genuine example of phlegmatia dolens. Dr. Davis has no scruple of this kind —nor indeed have we—but the Doctor certainly turns it to account in a manner rather too *forensic*. As the case itself, and Dr. Davis's commentary on it are short, we shall lay them before our readers.

"AN ŒDEMATOUS FOOT, FROM A COMPRESSURE OF THE CRURAL VEIN."

"'A woman, nearly thirty years of age, after a difficult labour, and in consequence of careless conduct, suffered much disturbance of her lochia. Her right leg was seized with an œdematous swelling, which extended from the groin to the heel, and enlarged the right labium pudendi. At the same time she was also seized with a loss of appetite.

"'Every probable means afforded by the art of healing was tried to remove the swelling, but without success. Neither diaphoretics, nor purgatives, nor diuretics, gave any relief; and fomentations and frictions excited the most violent pain. An incision was made through the cutis of the thigh, that the water might be drained off by an issue; but only a few small drops were discharged by it. The serum, in the cellular membrane, assumed in some sort the nature of a tremulous gelatine; all the more fluid part of it being resorbed. At the end of two months the patient died asthmatic.

"'On dissecting the body, we found some of the inguinal glands scirrhous, greatly enlarged, and surrounding the crural vein, by which its diameter was very much diminished.'"

DR. DAVIS'S COMMENT.

"In an analysis of this dissection, it is important to distinguish between the facts that are reported, and the opinion of the writer as to the order of their relation to each other as cause and effect. The simple facts of the case are enlargement and induration of the inguinal glands, and a great diminution of diameter of the crural vein. That this diminution of diameter in the vein was the effect of the compression presumed to have been made upon it by the enlarged and indurated glands, is to be received as a matter *purely of opinion.* In admitting, therefore, the fact of a diminution of diameter in the vein, we are by no means bound by the author's opinion as to the cause. On the contrary, it is my firm belief, that the actual cause of the asserted diminution of capacity in the vein, was the effect of a primary disease of the vessel itself; and that the inguinal glands had become enlarged and indurated in consequence of their immediate vicinity to the original seat of disease in the crural vein." 425.

Now, we appeal to the impartial, whether Dr. Davis is justified in reversing the conclusion drawn by Zinn? To us it appears infinitely more probable that a cluster of enlarged and indurated glands in the groin should make pressure upon, and diminish the calibre of, the crural vein, than that a narrowing of the vein should enlarge and indurate the inguinal glands. That a special pleader at the bar should endeavour to give this version of the affair, for the good of his client, might not be wondered at, but we certainly think a pathologist weakens, rather than strengthens his cause by evidence and reasoning of the above description.

Dr. Davis now proceeds to the facts which have come under his own observation and that of a friend, Mr. Oldknow of Nottingham, premising that it is his object to prove that

"The proximate cause of the disease called plegmatia dolens, is a violent inflammation of one or more of the principal veins within and in the immediate neighbourhood of the pelvis, producing an increased thickness of their coats, the formation of false membranes on their internal surface, a gradual coagulation of their contents, and occasionally a destructive suppuration of their whole texture; in consequence of which, the diameters of the cavities of these important vessels become so greatly diminished, sometimes so totally obstructed as to be rendered mechanically incompetent to carry forward into their corresponding trunks the venous blood brought to them by their inferior contributory branches." 426.

Such is our author's theory of the proximate cause of phlegmatia dolens, and he next proceeds to adduce the proofs.

*Case* I. Caroline Dunn, aged 21 years, of weakly constitution, was delivered of a male child on the 7th February 1817, after a severe and protracted labour. On the following day there was soreness in the vagina, and some fever, which continued during the next six or seven days. On the 13th she had still fever, with inflamed, swelled, and œdematous labia pudendi, copious yellow discharge from the vagina, &c. She got better, however, and on the 22d was able to sit up.

" '26th. Worse: left leg and thigh much swollen; pain in the inguinal region, skin hot, no signs externally of inflammation, no pitting on pressure, bowels costive, slight cough, respiration difficult, pulse very quick and small, headache.

" 'Feb. 28th to March 2nd. No better:—leg pitted on pressure, countenance depressed, languor, giddiness at intervals, pulse 80, freedom from pain, no appetite, bowels twice relieved.

" '3d. Total insensibility:—limb equally swollen, countenance pale, sunk and emaciated.

" '4th. Died at noon this day." 428.

The body was examined by Mr. Lawrence, which is a sufficient guarantee for the correctness of the dissection.

The left lower extremity presented a uniform œdematous enlargement, without any external discoloration, from the hip to the foot. "This," says Mr. Lawrence, "was found to proceed from the *ordinary anasarcous effusion into the cellular substance.*" The inguinal glands were a little enlarged. "The femoral vein, from the ham upwards, the external iliac, and the common iliac veins, as far as the junction of the latter, with the corresponding trunk of the right side, were distended, and firmly plugged with, what appeared externally, a coagulum of blood. The femoral portion of the vein, slightly thickened in its coats, and of a deep red colour, was filled with a firm bloody coagulum, closely adhering to the sides of the tube, so that it could not be drawn out. The trunk of the profunda was distended in the same way as that of the femoral vein; but the saphena and its branches were empty and healthy."

" The substance filling the external iliac and common iliac portions of the vein was like the laminated coagulum of an aneurismal sac, at least, with a very slight mixture of red particles. The tube was completely obstructed by this matter, more intimately connected to its surface than in the femoral vein; adhering, indeed, as firmly as the coagulum does to any part of an old aneurismal sac. But, in its centre, there was a cavity containing about a tea-spoonful of a thick fluid of the consistence of pus, of a light brownish red tint, and pultaceous appearance." 430.

Mr. Lawrence had no hesitation, of course, in pronouncing the above appearances to be the products of inflammation.

Before proceeding to the next case, we take the liberty of differing from Dr. Davis, on the identity of the case described with that of real phlegmatia dolens. We ground our first doubt on the *fatal issue* of the case, which is contrary to the general experience of the profession hitherto; for it must be recollected that Zinn's patient died of asthma, and not of phlegmatia dolens. If then there are very few cases on record where phlegmatia dolens in itself proved fatal; we have, at least, grounds for supposing, (we do not say that it amounts to proof) either that Dr. Davis's case was *not* phlegmatia dolens, or that its proximate cause was different from the proximate cause of phlegmatia dolens in general.*

Our main doubt, however, is grounded on the anatomical, or rather, pathological difference between Dr. Davis's case and those described by other authors. We have Mr. Lawrence's authority that the *enlargement* of the limb proceeded from *ordinary anasarcous effusion* into the cellular substance. Does this state harmonize with the description of phlegmatia dolens, as given by authors, or as seen by practitioners? It is contradistinguished from *anasarcous infiltration* in all the writers we have perused—(and certainly by our own observation, in at

---

* Is it likely that so serious, and generally fatal a disease as is inflammation of the internal coats of veins, under other circumstances should be almost invariably devoid of danger in phlegmatia dolens?

And here we shall introduce, from the 41st volume of the Dict des Sciences Medicales, a case which shews that *inflammation of the crural veins* has long ago been described after parturition, but without naming the disease phlegmatia dolens.

Sect. vi. *Inflammation des Veines à la Suite des Couches et de l'Avertement.* Meckel a publié, dans une Dissertation de Sasse, *Plusieurs faits d'Inflammation des Veines Crurales à la Suite des Couches.* Voici une observation que Schwilgué lui a emprunté. "Peu de temps s'etait ecoulé depuis la delivrance d'une femme, quand elle éprouva de la fièvre, des tiraillemens douloureux dans l'abdomen et dans le bassin, qui disparurent; mais au bout d'environ trois semaines, il survient une fièvre erratique, de l'expectoration, une douleur dans la region du foie, ainsi que dans la hanche guache, et une douleur intolerable dans la cuisse du même cotè. A l'examen du cadavre, on trouva la cavité abdominale remplie d'une matière purulente, le foie tres-volumineux, et les poumons sains. *Les vaisseaux cruraux etaient, ainsi que les nerves du même nom, entourés d'une matière puriforme; la veine crurale, examinée depuis son origine jusqu'au genou avait l'epaisseur et la consistance de l'artere;* ELLE ETAIT REMPLIE DE PUS ET DE SANG, *tandis que l'artere ne contenait que ce dernier liquide.* Les parois de la veine criaient sous le ciseaux; sa membrane interne etait plus spongieuse que dans l'etat ordinaire, et recouverte d'une fausse membrane très-distincte qui s'en laissait separer par lambeaux. Ses valvules etaient en partie corrodées, dechirées, et en partie epaissies, tumefiées et de couleur foucée." [*Schwilgue Memoire, cité p.* 19.]

In the above case, we have as complete a case of inflammation of the crural vein, as can possibly be cited; but the phenomena by no means correspond with those noted in phlegmatia dolens, with the mere exception of intense pain in the thigh.

least four or five cases) by the tense, or hard, or, at all events, *elastic* swelling of the limb—*not pitting* on pressure. What is Callisen's definition? "Tumor *elasticus*, albescens, renitens, calidus, dolens, foveam impressi digiti *haud retinens*." It is characterised by Dr. Dickson, of Plymouth Hospital, who has paid great attention to the subject, and who has written an excellent paper on the disease, as an "*unyielding*, white, glossy, swelling"—"incompressible," while "little subcutaneous knobs or prominences are often perceived on drawing the hand over its surface."

In a very well constructed paper on phlegmatia dolens, by the late Dr. Bateman, inserted in Rees's Cyclopœdia, and purporting, of course, to be drawn from the best authors, as well as personal observation, we have the following pointed expressions:—"the swelling is general and equal over the whole limb—it is much harder and firmer than in anasarca, in every stage of the disorder—it is not so cold, in any state of the disease, as the dropsical swelling; neither does it *pit* when pressed upon by the finger—nor does any water issue from it when it is punctured by the lancet." When these descriptions are compared and contrasted with Mr. Lawrence's dissection, we think every unprejudiced mind will agree with us, that Dr. Davis's case was of a character wholly different from genuine phlegmatia dolens. A case is also recorded by Dr. Denmark, of a disease resembling phlegmatia dolens, in a male; and his dissection of the case shews a disease, "the characteristics of which are strikingly different from those of œdema."

The second and third cases brought forward by Dr. Davis are very imperfect and unsatisfactory. But we shall give a fair epitome of them.

*Case* 2. A lady of sanguineous, irritable temperament, "died suddenly in the midst of apparently high and perfect health," on the 20th September 1819, six weeks after confinement. She had been seized with peritoneal inflammation the day after delivery, which yielded to active depletion. Ten days after this she made complaint of deep seated pain in the groin, and along the great vessels. Dr. D. found the limb swelled, and very painful; but, by leeches and blisters, "this new inflammation was speedily reduced," and, in a week, the "swelling had entirely subsided," the patient having recovered the perfect use of the limb. From this period she convalesced rapidly, and satisfactorily, dying, as before stated, in the midst of apparent health.

We apprehend that very few, on reading the above case abstractedly, would think of connecting it with phlegmatia dolens.

*Dissection.* In the thorax all was apparently sound. In the abdomen, there were adhesions between the viscera and the parietes, the consequence of previous inflammation. All the abdominal viscera themselves were healthy. Mr. Anderson and Mr. J. C. Taunton undertook the examination of the iliac veins.

"It is to them that I am indebted for the preparation, No. 2. It forms a part of the left external iliac vein, including about half an inch, of the upper portion of its corresponding femoral vein. That vessel was found strongly attached by adhesions of its cellular coat to the parts forming its natural bed. Its parietes still retained a morbid thickness, and its internal tunic was studded in several places with deposits of adherent lymph. The portion most remarkable for this incrustation, and otherwise most diseased, was the part of the vein immediately under Poupart's ligament. The appearance of that part is yet well preserved in the preparation, and forms the rough scabrous inferior portion of it. The tube of the vessel was still manifestly pervious, though it had suffered a diminution of capacity, amounting to, perhaps, one half of its natural diameter. The inguinal glands were not diseased." 435.

Of this case we can only say—valeat quantum valere debet.

*Case* 3. This case was communicated by Mr. Oldknow, of Nottingham. A woman was delivered in an easy and natural manner, in the month of September 1820. She did well for about three weeks. She was then seized with a violent diarrhœa, for which astringents were administered. Fever continued. On the 30th day from delivery, the purging returned, and "the left lower extremity became swollen and painful, with considerable increase of fever." Four days afterwards, she died.

*Dissection.* "'On examining the swollen limb the day after her death, I found the femoral vein, one third down the thigh, and all the iliac veins much enlarged, and containing adherent layers of coagulated blood, similar to that found in aneurismal sacs, together with a sort of grumous fluid of a brown colour, more or less mixed with air, and almost obliterating the venous canal. The same appearances, but in a much less degree, extended along the cava as far as the entrance of the renal veins. The coats of the veins were highly inflamed, and intimately attached to the surrounding parts. The absorbent vessels and glands were slightly enlarged as high as the lumbar regions, but not otherwise affected. The uterus had regained nearly its natural size.'" 436.

In the above, it will be seen that all the proof, during life, of phlegmatia dolens, "*is a swollen and painful lower extremity*"—and, in the dissection, not a word is said about the general state of the thigh and leg. The inflamed and obstructed vessels occupy the whole of the description. The patient died too on the *fourth day of the phlegmatia dolens.* Whether this case may be satisfactory to our readers we know not. To us, it conveys nothing decisive as to the pathology of phlegmatia dolens.

*Case* 4. A lady of delicate constitution and very irritable habit, was delivered on the 2d July 1821. She did well till the 7th day, when, being placed, apparently, in a current of air, she was seized with a violent rigor, and when reaction came on she was affected with intense pain in the left side of the chest. By decisive measures the pain in the chest was nearly subdued; but the fever continued. "In the evening of the same day, unequivocal symptoms of phlegmatia dolens declared themselves." This was on the 9th July. She died on the 23d of the same month.

On dissection, there was effusion and inflammation in the chest. "The left lower extremity, from the hip to the toe, was considerably but not greatly enlarged, and there was an evident fulness of the labium pudendi." The iliac veins on both sides were unusually turgid with blood. When the left was opened, it was found to contain a firm coagulum of blood, not adherent to the vessel at that place. Higher up, however, in the common iliac portion, the coagulum was adherent to the internal surface of the vessel. The left *internal* iliac was greatly inflamed, and its diameter so much contracted as to be almost impervious.

In the above case we have to regret that nothing is said of the state of the limb from the 9th July, when the "unequivocal symptoms" of phlegmatia dolens commenced, till the patient's death. In the dissection again, nothing is stated of the pathological condition of the limb. The whole attention is concentrated on the vessels. Now it ought to have been Dr. Davis's chief and main object to prove, in all these cases, that the disease was really phlegmatia dolens, by an accurate description of the symptoms and state of the limb, and then, to have traced the *cause*, if he could. But it is evident that the first and main object is almost totally neglected—or where it is adverted to, as in Mr. Lawrence's dissection, it makes against the question—and therefore we do not consider ourselves bound to subscribe to our author's etiology, without having the necessary documents respecting the symptoms and dissections of the cases.

That the inflammations and obstructions of vessels brought forward by Dr. Davis, would and did produce the tumefactions of the limbs, we entertain no doubt—but until Dr. Davis lays before the public a more circumstantial detail, we hesitate to acknowledge the cases in question to be genuine examples of phlegmatia dolens. We have, however, placed the *facts* before our readers—and they can judge for themselves, uninfluenced by our opinions on the subject. The author of the paper we have the pleasure of enrolling among our friends, and we know him to be an able teacher and excellent practitioner. But we know, also, that when a theory is to be established, *the brighter the genius the proner to error.*

Before adverting to the treatment of phlegmatia dolens, we shall give some account of the other papers whose titles stand at the head of this article.

Dr. Francis, of New York, has published an interesting *memoir* on this disease—many instances of which appear to have come under his notice both in females and males.

He considers the disease as varying in its causes and also its seat—not being confined to the lower extremities alone—nor to the female sex, nor to the period of parturition. In a case communicated to Dr. Francis, by Professor Macneven, the same individual was afflicted four different times, in four successive labours, "in the same limb," and "was characterized by all the diagnostic signs of the disease." He mentions a case in which Dr. Mann of Boston was consulted, and where, previous to his visit, the limb had been punctured, under the idea of a fluid being collected there. No discharge followed—the wound sphacelated, and the patient died. We shall give the following curious case of the disease in a male, when in the upper extremity.

"Dr. Heermans, of Ontario county, state of New-York, in a letter to Dr. J. B. Beck of this city, has detailed the history of a case of phlegmatia dolens in a young man aged nineteen; and so far as a single instance can be brought to militate against a general rule, it furnishes conclusive evidence that the superior as well as inferior extremities may become the seat of this disease. The patient was subject to rheumatic affections, and had been exposed to inclement and rainy weather some days before his illness. The symptoms of the disorder first exhibited themselves in the calf of one leg, and rapidly extended to the groin, with increase of pain and inability to move. His sufferings were so acute that he was unable to bear the slightest pressure or contact with the skin. At the lapse of thirty-six hours the limb was enormously distended, and had acquired the glabrous aspect and other pathognomonic symptoms of this striking affection. A similar swelling soon commenced in the other extremity; it began at the groin and descended in this leg as rapidly as it had ascended in the other, with the same sensation and appearances. The swelling now continued in both legs down to the extremities of the toes. On the fourth or fifth day, according to Dr. Heermans, the patient complained of the same kind of pain and swelling of the paits about one of the shoulders; but it did not diffuse itself with the same violence and rapidity as it had done in the lower extremities; in like manner the other superior extremity was assailed; after ten days from the first attack, the swelling and distress began to subside in the order in which they commenced; with the exception of the left arm, which continued distended seven or eight weeks, before it was reduced to its natural size. The disease was treated by active depleting remedies, frictions, and fomentations." 9.

In the 2d Number of the same Journal, there is a case by Dr. Beck, where phlegmatia dolens occurred in a woman 52 years of age. "The limb was tense,—shining, elastic, and exceedingly painful. No œdema was discoverable in any part of it." The patient informed Dr. Beck that, the disease had commenced with a feeling of deadness in the toes, heel, and upper part of the foot, which was shortly succeeded by severe pains in the part, after which the foot began to swell. "By the succeeding day the swelling had ascended to the knee, from which it gradually proceeded to the groin." The complaint yielded to depletion.

Dr. David Hosack has also seen a considerable number of cases of phlegmatia dolens, and relates nine cases in the Journal from which we are now quoting. We cannot stop to extract any of the details—except the notice of one case, which our author states to have first given him an enlarged view of the pathology of the disease. The lady had undergone a severe labour with twins, and had been confined 23 days, when

"She was first affected by cold producing catarrhal and pneumonic inflammation; but within forty-eight hours a metastasis took place to the limb, which proceeded to swell with all the symptoms of idiopathic cruritis, the affection of the lungs totally disappearing. By general blood-letting, saline cathartics, antimonials, tepid applications to the part affected, with a strict antiphlogistic diet and regimen, the first stage of the disease was in a few days removed; afterwards, by stimulating liniments, frictions, and the roller, the parts affected were restored." 54.

We shall give Professor Hosack's general conclusions drawn from a careful revision of the cases that came under his notice.

"1st. That cruritis is an inflammatory disease, not only affecting the limb, but the whole system.

"2d. That it most usually proceeds from a suppression of the natural excretions, the effect of cold, stimulating drinks, and other means of excitement.

"3d. That it is not necessarily connected with the lochial discharge, as inculcated by Trye, Denman, and indeed by Rodrigus Decastro, of

Hamburgh, in 1603, by Wiseman, in 1676, and by Mauriceau, in 1712, who were the authors of this doctrine.

"4th. That the first irritations frequently appear about the calf of the leg, and not in the groin and pelvis, as asserted by Dr. Denman.

"5th. That it follows easy as well as difficult labours, and therefore cannot proceed from the pressure of the child's head upon the edge of the pelvis rupturing the lymphatics, as supposed by Mr. White.

"6th. That it is not a disease confined to the lymphatics, but as in the cases recorded by Dr. Hull, it appears in every part of the affected limb.

"7th. That it is not confined to females, but, as in the cases recorded by Dr. Hull, Dr. Ferriar, Dr. Thomas, and others, it occasionally appears in males.*

"8th. That, as in gout and rheumatism, when depletion is not actively employed, the inflammation, after appearing in one limb, is in some cases transferred to another.

"9th. That it sometimes appears in both limbs at the same time.

"10th. That the general means of subduing inflammatory action are the most effectual in removing the *active* stage of this complaint.

"11th. That in the second stage of cruritis, in addition to the use of general stimuli and tonics, stimulating spirituous liniments, friction, and the roller, are most useful in restoring the circulation, and in exciting the absorbents in the removal of the swelling which remains in the passive stage of this disease.

"12th. That occasionally, as in the cases related by Hull, Denman, and by Zinn, it ends in abscess, and proves fatal, especially where the antiphlogistic treatment has not been vigorously pursued in the first stage of the disease, or when it occurs under great exhaustion and debility of constitution." 57.

After all that has been written on the pathology of phlegmatia dolens, and from what we have seen of it ourselves, we are more inclined to adopt the opinion of Dr. Hull than that of any other writer—namely, that it consists of a peculiar inflammation seated in the muscles, cellular membrane, and inferior surface of the skin, producing a rapid effusion of coagulable lymph from the exhalents into the cellular membrane of the limb. The obstructions or other organic changes which have been, or may be, found in the veins or lymphatics, we consider as secondary effects or contingencies; and cannot view them as the primary causes of the disease.

This naturally leads to the treatment. Dr. Davis condemns general bleeding, although the constitutional pyrexia, might seem to demand or authorize that measure. Experience, the

"* See Medical and Chirurgical Journal for 1817. Medical and Chirurgical Transactions for 1819."

best test of theory, has shewn him the inefficiency of venesection in phlegmatia dolens. Puzos was a strenuous advocate for general bleeding; but the practitioners of this country, who have written on the disease, have generally contented themselves with local bleeding, and moderate antiphlogistic regimen, in the early or inflammatory stage of the complaint. There can be no doubt, however, that cases may occur, where general depletion may be rendered necessary, by the extent of the general fever; and, therefore, venesection should not be totally proscribed from the methodus medendi.

"I am happy," says Dr. Davis, "to have it in my power to assure the Society, that the great indication of treatment in this disease, as already proposed; viz. the speedy resolution of the inflammation in the iliac veins, is to be secured in almost every case (I have not seen an exception) by early and decisive local treatment. The blood to be abstracted should, accordingly, be all taken from the immediate neighbourhood of the part primarily affected. From the excessive tenderness of the parts concerned, leeches are the only operators to be depended upon in these cases. Of these, a dozen, or a dozen and a half, should be forthwith applied to the groin, to the affected iliac region, and to the interior and superior part of the thigh. If this be done before any very obvious accumulation of blood in the limb shall have taken place, it will generally put down the threatened mischief at once. In the event of our first success proving incomplete, a large blister should be applied to the groin and parts adjacent, both above and below. These measures are to be varied and repeated according to the particular circumstances of the case." 456.

During the progress of the swelling, and accompanying evolution of heat, the limb should be cooled, and kept at a low temperature, by evaporating lotions, and exposure to the action of the atmosphere. To this practice Dr. D. attaches much importance. This treatment corresponds with what our able and judicious friend Dr. Dickson has laid down in the number of this Journal for July 1819. "The most successful mode of treatment," says he, "consists in the free and early use of leeches—in purgatives—cloths wetted with tepid fluids to abstract morbid heat—saline and antimonial medicines, according to the degree of fever—quietude and horizontal posture—and the pulvis ipecac. comp. or other occasional opiates to allay pain, or irritation." Dr. Dickson properly observes that—"where the patient is of a full, strong habit, with considerable symptomatic fever, it will be proper to take blood from the arm previously, and to enforce the antiphlogistic regimen." Dr. D. considers local bleeding, by leeches, as generally sufficient, especially if applied early, and in sufficient numbers. Twelve or eighteen should be applied to the groin, at the onset of the disease, and a smaller number repeated lower down, according to circumstances. It is hardly necessary to observe that, when the inflammatory symptoms have subsided, it will be proper to apply bandages, and stimulating applications to the surface. while tonics are exhibited internally.

Dr. Davis observes, that he is not aware of having derived any advantage from the exhibition of antimonials in this disease. Where it has been his objeet to reduce arterial action, and when he has met with cases of more than ordinary obstinacy, he has, of late years, had recourse to digitalis, in full and frequent doses—"viz. in doses of two grains of the powder (Battley's preparation) every two, or at furthest, every three hours."

"My experience of this mode of exhibiting the digitalis in acute diseases, enables me to state, with confidence, that it may be safely administered to adults, at such intervals, and in such quantities, until the patient shall have taken from twenty-five to thirty grains of it. It should then be proceeded with more slowly, until some one or more of its peculiar effects on the nervous system, or the circulation, be produced, when it should be immediately suspended; to be again resumed, or not, according to circumstances. It will generally be an advantage to keep the circulation under its controul, for several weeks, as an insurance against the accession of the disease in the other extremity. I need not observe that the foxglove is a potent drug, and that it requires much caution, and constant watching in its exhibition. I generally combine it with a small quantity of the blue pill; which, I think, prevents it, in a great measure, from nauseating the stomach. I do not approve of the use of active purgatives in this disease. The bowels, of course, should be kept moderately open, as, indeed, should all other important functions of the system, be placed under due and well-balanced regulation." 468.

In conclusion, we beg to say that, in differing in opinion with Dr. Davis on certain pathological points, we have done so with reluctance, as none can entertain a sincerer respect for that gentleman's talents, zeal, and attainments, than we do. But, conceiving it to be our duty and our right, to offer our sentiments with candour and freedom on the passing theories or practices of the day, we shall do so with diffidence; but, we trust, also, with becoming firmness. We have no need to flatter—and we have no wish to offend.

